# An Organ System Approach to Explore the Antioxidative, Anti-Inflammatory, and Cytoprotective Actions of Resveratrol

**DOI:** 10.1155/2015/803971

**Published:** 2015-06-09

**Authors:** Ashim Malhotra, Sundeep Bath, Fawzy Elbarbry

**Affiliations:** School of Pharmacy, Pacific University, 222 SE 8th Avenue, Suite 451, Hillsboro, OR 97123, USA

## Abstract

Resveratrol is a phenolic phytochemical, with a stilbene backbone, derived from edible plants such as grape and peanut. It is a bioactive molecule with physiological effects on multiple organ systems. Its effects range from the neuroprotective to the nephroprotective, including cardiovascular, neuronal, and antineoplastic responses as a part of its broad spectrum of action. In this review, we examine the effects of resveratrol on the following organ systems: the central nervous system, including neurological pathology such as Parkinson's and Alzheimer's disease; the cardiovascular system, including disorders such as atherosclerosis, ischemia-reperfusion injury, and cardiomyocyte hypertrophy; the kidneys, including primary and secondary nephropathies and nephrolithiasis; multiple forms of cancer; and metabolic syndromes including diabetes. We emphasize commonalities in extracellular matrix protein alterations and intracellular signal transduction system induction following resveratrol treatment. We summarize the known anti-inflammatory, antioxidative, and cytoprotective effects of resveratrol across disparate organ systems. Additionally, we analyze the available literature regarding the pharmacokinetics of resveratrol formulations used in these studies. Finally, we critically examine select clinical trials documenting a lack of effect following resveratrol treatment.

## 1. Introduction

**Figure 1:**
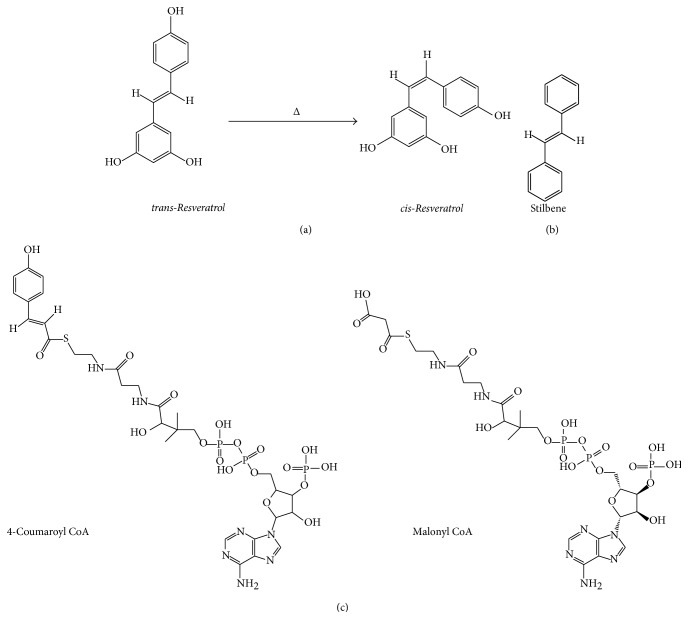
The chemistry of resveratrol. (a) shows the* trans*- and* cis*-isomers of resveratrol. (b) shows the stilbene C6-C2-C6 backbone, which is chemically modified in resveratrol. (c) describes the overall biosynthetic process in plants, beginning with a reaction of coumaroyl-CoA and malonyl-CoA catalyzed by stilbene synthase.

Resveratrol is a polyphenolic phytochemical that is biosynthesized by certain edible plants such as grape, peanut, and berry in response to phytogenic insults or pathogens. As shown in Figure 1, chemically it is 3,5,4′-trihydroxystilbene, a stilbene derivative, which is a phenylpropanoid with a C6-C2-C6 general structural formula. It is found as two isomeric structures in nature, the* cis*- and* trans*-isoforms, the* trans*-isoform being convertible to* cis*-isoform by heating. Its biosynthetic pathway begins with coumaroyl-CoA and 3-malonyl CoA and involves the enzyme stilbene synthase, which leads to a general biological yield of 50 to 400 *μ*g resveratrol per g wet weight of leaves [1, 2]. Since it is biosynthesized to a greater extent in the lignified parts of these plants, the process of crushing and mashing grapes for the purpose of wine making results in relative enrichment of resveratrol in wine, making the latter a significant dietary source of resveratrol. The high resveratrol level in red wine was postulated as a factor in the “French Paradox”, where epidemiological data revealed an apparent disconnect between French patterns of low rates of cardiovascular disease despite their high saturated fat consumption. Interestingly, based on its function in plants, resveratrol may be classified in the general category of low molecular weight compounds known as phytoalexins, which are phytochemicals possessing an antimicrobial and antioxidant function.

However, in the context of therapeutic engagement, resveratrol exhibits a problem shared by many other potential drugs, that of its hydrophobicity, which ameliorates its bioavailability. Indeed its pharmacokinetic properties, discussed in detail later, engender a short residence time in the body, making this an unlikely therapeutic agent due to pharmaceutical and bioavailability issues. However, taking into consideration its overall multifaceted biological activity that ranges from anti-inflammatory to cardio-, neuro-, and nephroprotective effects and to antineoplastic attributes, a lot of innovative work has gone into the design and formulation of nano- and microemulsion-based formulation systems for resveratrol. These pharmaceutical formulation techniques enhance the bioavailability of this drug and prolong its duration of action. Neves et al. have provided a detailed review and analysis of some of these resveratrol formulations and the interested reader is referred to this work [1].

This review focuses on providing a broad overview of the experimental studies and clinical trials conducted to analyze the systemic anti-inflammatory, antioxidative multiorgan protective effects of resveratrol. We substantiate this information by summarizing molecular pharmacology mechanisms and specific second messenger pathways, along with pharmacokinetic data with reference to resveratrol.

## 2. SiRT1 and Resveratrol: Heading towards a Centralized Mechanism

Sirtuins are a family of proteins involved in a number of processes in cells, but some are particularly associated with cellular and organismal longevity. Multiple mammalian isoforms, SIRT 1–7, exist, with SIRT1 being the molecular target of small molecule activators such as resveratrol, which can induce its expression by 10-fold. Generally induced by restricted calorie diet, these molecules have been proposed to be the molecular connecting link between the physiological effects of extraneous chemicals and subsequent genetic responses in a variety of tissue and cell types across various organs. Although Howitz et al. suggested that SIRT1 was the intracellular target of resveratrol [11], there was considerable controversy about this claim, as later studies conducted by two groups, Kaeberlein et al., and Borra et al. seemed to suggest that activation depended on the fluorescent moiety (aminomethylcoumarin, or AMC) attached to the peptide substrate in the assay [12, 13]. However, later mice experiments using gene knockout technology confirmed not only the role of SIRT-1, but also the role of its downstream second messengers such as AMPK [14]. Thus, it appears that in many of the tissues tested, such as the kidney or cardiovascular tissue, SIRT1 is the molecular switch through which resveratrol mediates its effect.

## 3. Resveratrol in Kidney Disease

In the field of nephrology, resveratrol has shown great promise in ameliorating the effects of renal cytotoxicity in animal-based experiments. In general, renal disease may be classified as acute or chronic, each with multifactorial etiology, and may be of primary or secondary causation depending on whether the kidney is the major site of origin. For instance, nephropathy resulting from complications of uncontrolled diabetes over a long period of time may be considered a secondary manifestation, whereas glomerulonephritis due to a toxicant, which may lead to acute nephropathy, may be classified as a primary disease. In addition to extraneous agents, the nephropathy may arise from genetic or metabolic causes, such as the development of kidney stones and some forms of polycystic disease. Interestingly, a body of work developed over the past decade documents the advantage of using resveratrol in alleviating symptomatology or the pathological progression of many of the renal disease states introduced above.

Acute kidney injury (AKI) may become the precursor to chronic disease, if the toxicant remains in prolonged contact with the kidney or the pattern of pathological lesions becomes recurrent. In these situations, AKI may lead to renal fibrosis, which in turn may eventually lead to renal failure. Although kidney function may linearly deteriorate with progressive renal disease, clinically significant gains may be made by the therapeutic reversal of fibrosis and prevention of further damage to the tissue. Alterations in the renal microenvironment, such as those that occur as a consequence of advancing renal disease, generally manifest as altered expression of extracellular matrix (ECM) proteins in the affected tissue [15]. Resveratrol has been shown in rat models of renal fibrosis to effect changes in ECM protein expression. Liang et al. examined the effects of resveratrol at 20 mg/kg/day in murine experiments, where experimental renal fibrosis was induced by unilateral ureteral obstruction. The study showed resveratrol to be effective in decreasing the renal cortical mRNA levels of the ECM proteins, specifically the cell adhesion molecules, fibronectin, and ICAM-1. Additionally, it also reduced TNF-*α* and TGF-*β*, which may be involved in renal inflammation and the production of reactive oxygen species (ROS) such as malondialdehyde (MDA) and 8-hydroxydeoxyguanosine (8-OHdG) [16]. Detailed sequence of the underlying molecular mechanism resulting in reduced TGF-*β* expression following resveratrol treatment was elucidated in a study by Huang et al. Using cultured mesangial cells and kidney pieces from nephrectomized rats, these authors demonstrated that the nephroprotective effect of resveratrol was mediated by the activation of SIRT1, a member of the sirtuin family of proteins capable of downregulating p53, which resulted in an inhibition of the TFG-*β*/Smad3 pathway [15].

As introduced above, renal damage may also occur as a consequence of other primary diseases such as diabetes. Resveratrol ameliorated nephropathy in a rat model of streptozotocin-induced diabetes, normalizing creatinine and adiponectin levels and reducing polyuria and proteinuria. This was mediated through counteracting ROS activity via augmenting enzymatic activities of superoxide dismutase and glutathione reductase and enhancing the expression of the transcription factor nuclear factor erythroid factor 2 (Nrf2) [17, 18]. A number of independent studies further explored the molecular mechanisms of resveratrol-mediated nephroprotection. Ding et al. showed that resveratrol delayed the progression of diabetic nephropathy by activating AMPK signaling, while suppressing phosphorylation of 4E-binding protein 1 (4E-BP1) and ribosomal protein S6 in diabetic rat kidneys [19]. Wu et al. demonstrated increased SIRT1/FOXO1 activity in the diabetic rat kidney, effectively attenuating renal oxidative stress [20].

The nephroprotective effects of resveratrol were not limited to an amelioration of pathological renal fibrosis but extended to other renal morbidities such as nephrolithiasis, also known as the deposition of kidney stones. Hong et al. investigated the effect of resveratrol in rats with ethylene glycol-induced kidney stones. Resveratrol exhibited antinephrolithic properties by both inhibiting free radical formation and attenuating the expression of a number of inflammatory mediators and ECM proteins such as TG*β*-1, osteopontin, hyaluronan, and MCP-1 [21].

In summary, resveratrol has shown protective effects against several types of kidney injuries mostly due to its antioxidant effect and activation of SIRT1. Further studies should be undertaken to examine the potential effect of resveratrol in human and animals models of kidney injuries.

## 4. Resveratrol in CNS Disease

The molecular pharmacology of resveratrol in the central nervous system follows the general themes for other organ systems, in that it produces a wide spectrum of physiological responses by affecting diverse functions. Equally interesting is the orchestration of second messenger systems in the brain, which parallels the activation of similar, if not the same, second messengers in other organs, following resveratrol treatment. It is interesting to note that a polyphenol phytochemical such as resveratrol elicits an influence over disparate neurological phenomena ranging from developmental neuronal processes to those involved in aging and dementia, probably by acting as a small molecule regulator of multiple intraneuronal processes.

The past two decades have seen tremendous effort in the elucidation of the molecular mechanisms of progressive dementia such as Alzheimer's disease (AD), with multiple breakthroughs in molecular research leading to novel possible therapeutic interventions. Approaching AD from the perspective of protein misfolding and dysfunction has shed new light on pathological mechanisms. For instance, the “tau” protein is important for mitochondrial transport and therefore energy production in axons; its phosphorylation is associated with the progression of AD. Tau acetylation is increased in early to moderate Braak stages of tauopathy, which describe the degree of neurofibrillary tangles (NFTs) involved in AD. Therefore, modulating tau acetylation can serve as an effective new target in ameliorating neurodegeneration in relation to AD. Min et al. demonstrated that* in vitro* treatment with resveratrol leads to modulation of tau acetylation and thus attenuated tau-mediated neurodegeneration [22]. Additionally, in the context of AD, resveratrol was demonstrated to enhance the intracellular degradation of *β* amyloid peptides through a mechanism that implicated the proteasome [23]. Extracellular accumulation of *β* amyloid plaques was identified as an early pathological lesion in AD development. The beneficial effects of resveratrol for AD were not limited to reduction of *β* amyloid plaque but in a different study resveratrol improved mitochondrial function, reduced ROS activity, and promoted neuronal cell survival via activating the SIRT1 pathway [24]. As noted, involvement of SIRT1 downstream of resveratrol is a feature shared by the resveratrol-mediated intracellular protection pathway in multiple organs, for instance as discussed above for the kidney.

Telomeres are located at the end of chromosomes and become shorter in length with cell division. Telomere shortening is correlated with aging and is believed to play a key role in the pathogenesis of AD. Shorter telomeres are associated with increased neuronal cell death, as they are more susceptible to stressors such as ROS and UV radiation. Resveratrol mediated neuroprotective effects* in vitro*, by promoting the expression of telomere-maintenance factors such as WRN helicase gene [25]. Additionally, the neuroprotective effects of resveratrol included anti-inflammatory regulation of *β* amyloid-triggered microglial activation. Microglial cells serve a macrophage-like scavenger function in the CNS and remove infected and dying cells by phagocytosis. The anti-inflammatory effect of resveratrol is associated with the inhibition of the nuclear factor *κ*-light chain enhancer of activated B cells (NF*κ*B), STAT, and TLR4 signaling pathways [26].

Neuronal inflammation may also be associated with other CNS disease states. For instance, inflammatory mediators and activation of glial cells seem to contribute to dopaminergic cell death in Parkinson's disease (PD). Such selective neurotoxicity, followed by apoptosis, is considered to be hallmark of the pathogenesis of PD, which is believed to originate from an imbalance in acetylcholine and dopamine in the CNS. The protein, suppressor of cytokine signaling (SOCS-1), regulates several CNS processes including glial cell activation and inflammatory reactions. The neuroprotective action of resveratrol was observed in murine models of Parkinson-like disease, where treatment with this polyphenol resulted in the expression of SOCS-1, effectively impairing microglial activation and inflammatory reactions [27]. Further, resveratrol administration to PD rats at 20 mg/kg/day for 14 days resulted in a reduction in all the following: (1) abnormal rotational behavior [28], (2) nigral cell death, and (3) ROS species [27]; the first two are directly linked to the phenotypic manifestation of PD in humans and may offer relief from debilitating tardive kinesis and dyskinesia. In this experimental model, liposomal delivery of resveratrol augmented its neuroprotective effects [28]. Finally, oxidative stress is one of the mechanisms thought to induce apoptotic death of dopaminergic neurons in PD. Resveratrol treatment ameliorated the effect on MPP+ (Parkinsonian toxin) induced oxidative stress on dopaminergic neurons. Pretreatment with resveratrol diminished neuronal cell death by modulating pro- and antiapoptotic proteins such as Bax and Bcl-2 [29].

In addition to anti-inflammatory effects, resveratrol induced the production of neurotrophic factors, such as the glial cell line-derived neurotrophic factor (GDNF) and brain derived neurotrophic factor (BDNF), which play an important role in the survival of neurons and oligodendrocytes. Neurotrophic factors originate from the astroglia and may be a promising therapy for neurodegenerative diseases like PD. Resveratrol was effective in increasing production of neurotrophic factors in astroglial-enriched culture mediums, four- to sixfold, compared with control cultures. It was postulated that resveratrol mediated the release of neurotrophic factors via the activation of extracellular signal-regulated kinase 1/2 (ERK1/2) and cAMP responsive element-binding protein (CREB) in the astroglia [30].

In relation to other types of neurological stressors, the recent literature has found a strong correlation between hyperglycemia and neurodegeneration. Resveratrol ameliorated the effects of hyperglycemia-induced oxidative stress and subsequent apoptosis of dopaminergic neurons, by regulating the genes modulating the apoptotic cascade and reducing p53 and superoxide anion expression [31].

Amyotrophic lateral sclerosis (ALS) is a progressive neurodegenerative disease characterized by selective loss of motor neurons in the brain and spinal cord. Using a mice model of ALS, resveratrol was found to delay the disease onset, extend life span of animals, ameliorate loss of motor neurons, and inhibit mitochondrial dysfunction and muscle atrophy [32, 33]. Such beneficial effects of resveratrol were associated with enhanced expression and activation of SIRT1 and AMPK in vertebral spinal cord, improved lipid peroxidation, and inhibition of P53 and its downstream apoptotic pathway.

In summary, the above* in vitro* and animal studies provide growing evidence that resveratrol has beneficial effects in several neurodegenerative diseases. These effects are mostly attributed to its antioxidant effect, activation of SIRT1, and anti-inflammatory effect. Although many clinical trials have been conducted recently to investigate the potential effect of resveratrol in neurodegenerative disorders, there is no complete large scale clinical trial coming to a conclusive evidence [34, 35].

## 5. Resveratrol and Cancer

Resveratrol is similar to many bioactive phenolic phytochemicals in eliciting an antineoplastic response. Its antineoplastic properties may be classified into three broad areas: (1) antiproliferative, (2) antitumorigenic, and (3) antimetastatic. Though the following studies detail the specific molecular mechanisms of action, which may differ slightly, the sheer breadth of experimental cancer models and cancer cells that resveratrol is active against provides compelling evidence for conducting clinical trials in humans and for advocating its use in combination chemotherapy as a possible adjuvant. However, a careful analysis of its mechanism of action must be completed before any such endeavor is instituted, as exemplified by the estrogenic studies done on resveratrol that are outlined below. Interestingly, overall resveratrol treatment greatly benefits carcinoma therapy, suggesting the involvement of similar intracellular molecular signaling pathways downstream of resveratrol treatment that result in tumor regression in carcinomas.

### 5.1. Resveratrol and Cancer Pathogenesis

#### 5.1.1. Colon Cancer

Resveratrol has been found to possess anticancer activity against colon cancer both in human clinical trials and in experimental animal models in the laboratory.

Nguyen et al. conducted a phase I pilot clinical trial examining the effect of plant-derived resveratrol and grape powder on Wnt gene expression in colonic mucosa and colon cancer. The Wnt signaling pathway is implicated in the pathogenesis of colon cancer, with activating mutations seen in over 85% of cases. This clinical trial involving colon cancer patients showed that resveratrol administration reduced Wnt gene expression in normal mucosa cells but had no effect on cancerous cells [36].

In an experimental rat model, Tessitore et al. supplemented the drinking water supply to a final resveratrol dose of 200 *μ*g/kg/day and demonstrated significant reduction in large aberrant crypt foci (ACF) in rat colons. ACF are clusters of abnormal glands that line the colon and rectum and form before the development of colorectal polyps. The regulation of Bax and p21 is pertinent to the pathogenesis of colon cancer since Bax is a proapoptotic member of the Bcl-2 family and p21 is a cyclin-dependent kinase inhibitor and thereby involved in cell cycle progression. The protective role of resveratrol in colonic carcinogenesis was achieved by modulating Bax and p21 expression in the ACF but not in the rest of the colon mucosa [37].

#### 5.1.2. Skin Tumors

In a similar vein, treatment of mice with resveratrol led to a decline in 7,12-dimethylbenz[∝]anthracene- (DMBA-) initiated skin tumors. Resveratrol was moderately effective in reducing DMBA induced tumor formation through induction of apoptosis, which was mediated via upregulation of p53, Bax, and apoptotic protease activating factor-1 (APAF-1), cytochrome c release from the mitochondria, and inhibition of Bcl-2 [38, 39].

Ultraviolet radiation is a major cause of nonmelanoma skin cancer and resveratrol treatment; both pre- and post-UVB exposure in mice resulted in a decrease in skin tumor formation. Resveratrol modulated its UVB protective effects via suppressing mRNA levels of protein survivin and increasing proapoptotic proteins like Smac [40].

#### 5.1.3. Breast Cancer

Breast cancer is another area of oncology research where resveratrol treatment has shown beneficial responses in experimental models and where direct evidence of the molecular mechanisms of resveratrol has accumulated over the years.

Banerjee et al. showed that treatment of mice with resveratrol at dietary levels led to a reduction in the formation of DMBA-induced mammary tumors and effectively prolonged the latency period in mice. Its antitumorigenic effects were elicited through an inhibition of COX-2, matrix metaloproteinase-9 (MMP-9), and NF*κ*B activation in breast tumor, since the enzymes MMP-9 and COX-2 are involved in the tumor metastasis, and NF-*κ*B mediates tumor cell proliferation [41]. However, in addition to downregulating these enzymes, Bhat et al. [42] demonstrated heterogeneity in resveratrol-mediated estrogenic engagement using disparate experimental models of breast cancer. Bhat and colleagues demonstrated that resveratrol possessed both weak estrogenic potential as well as strong antagonism when coadministered with estrogen to breast cancer cells. The cell type appeared to influence the ratio of agonism to antagonism of resveratrol, indicating a tissue specific response, suggesting that resveratrol behaved as a selective estrogen receptor modulator or SERM drug. Further, by inducing apoptosis, suppressing angiogenesis through reducing VEGF extracellular levels and modulating progesterone receptor expression, resveratrol supplementation in mice positively impacted breast cancer development [43]. Provinciali et al. demonstrated that supplementation of the drinking water of experimental mice resulted in a reduction of mammary tumor formation and decreased the average number and size of mammary tumors. In the context of cellular mechanisms, these findings were associated with a downregulation of HER-2/neuexpression and increased apoptosis in mammary gland tumors and in other tumor cell lines [44].

#### 5.1.4. Prostate Cancer

Mechanistic analysis of the action of resveratrol in prostate cancer was carried out in different studies. Harper and colleagues used a dose of 625 mg resveratrol per kg body weight and showed that resveratrol suppressed prostate cancer progression in a male transgenic adenocarcinoma mouse prostate cancer model. Resveratrol mediated chemopreventive effects against prostate cancer by reducing cell proliferation, insulin-like growth factor-1, and simultaneously downregulating the phosphorylated and activated MAP kinases called extracellular regulating kinase, ERK-1 and ERK-2 [45], which are involved in pathogenesis of prostate cancer [45]. In transgenic rats, Seeni et al. found that resveratrol inhibited prostate tumor growth via modulating apoptosis through androgen receptor downregulation and inhibiting androgen responsive glandular kallikrein (an ortholog of human prostate specific antigen) [46].

#### 5.1.5. Hepatocellular Carcinoma

Resveratrol induced apoptosis of tumor cells in rats modeling hepatocellular cancer via facilitating an increase in cells in the G2-M phase and upregulating Nrf2 expression [47, 48]. According to Carbó et al., resveratrol administration resulted in a 25% reduction in tumor cell content [47]. Furthermore, Bishayee et al. noticed that resveratrol supplementation at 100 and 300 mg/kg decreased the appearance and number of hepatocyte nodules in Sprague-Dawley rats. In this study, the effect of resveratrol was evaluated in hepatitis B infected mice, which later spontaneously developed hepatocellular carcinoma (HCC) [48]. Resveratrol administration at 30 mg/kg/day for 4 months in transgenic mice significantly decreased the incidence of hepatitis B virus-mediated HCC by 5.3-fold. Resveratrol suppressed ROS production and enhanced hepatocyte proliferation, thereby replacing damaged cells in the transgenic liver and promoting regeneration. Furthermore, hepatocyte proliferation returned to basal levels after 30 days of resveratrol supplementation when mice recovered from the liver pathology [49].

#### 5.1.6. Pancreatic Cancer

Cancer of the pancreas may originate from a variety of cellular sources within the pancreas, but the more common is pancreatic adenocarcinoma which, like the carcinomas of the breast and the prostate gland discussed above, originates from the ductal epithelium. Resveratrol caused a decline in pancreatic tumor growth in rats in a dose-dependent manner [50, 51]. The antitumor effects of resveratrol were linked to decreased activity of the inflammatory enzyme leukotriene A4 hydrolase and a promotion of cell cycle arrest through the activation of the FOXO transcription factor [50, 51]. Resveratrol was successful in inducing apoptosis in pancreatic cancer cells* in vitro*, through suppressing mIR-21 expression which is linked with the downregulation of Bcl-2 expression [52].

#### 5.1.7. Gastric Cancer

The physiological effects of resveratrol were also evaluated in congenic mice. Congenic mice provide an experimental animal model that contains mutated adenomatous polyposis coli (APC) genes, as a result becoming predisposed to developing intestinal tumors. Resveratrol supplementation in congenic mice led to a 70% decline in small intestinal tumor formation through the downregulation of genes involved in the cell cycle progression such as cyclins D1 and D2 and the DP-1 transcription factor. Furthermore, resveratrol promoted suppression of the carcinogenesis process by activating cytotoxic T cells, leukemia inhibitory factor receptor, and monocyte chemotactic protein-3 [53]. Resveratrol inhibited the growth of human primary gastric carcinoma cells in nude mice, which lack the ability to mount an immune response, by promoting apoptosis via attenuating the expression of* BCL-2*, while enhancing* BAX* gene expression [54].

#### 5.1.8. Lung Cancer

Resveratrol treatment reduced tumor volume (42%) and weight (44%) in a lung cancer model at doses of 2.5 and 10 mg/kg. Resveratrol also reduced metastasis (56%) by significantly decreasing the number of tumor cell colonies that metastasized to the lung in Lewis lung carcinoma bearing mice [55]. The authors suggested that the antitumor activities of resveratrol might be linked to increased apoptosis via reduction in the S phase population and inhibition of neovascularization [55]. Furthermore, Lee et al. also noticed antitumor effects of resveratrol via caspase-mediated apoptosis, increased expression of caspase-9 and caspase-3, and suppression of tumor proliferation through inhibition of basic fibroblast growth factor (BFGF) [56].

Combination therapy may be an effective approach in the treatment of cancer. Radiotherapy is one of the most widely used interventions in cancer treatment; however, its success is limited by the development of resistant cancer cells. Thus, using resveratrol as an adjuvant drug, along with the use of increased ionizing radiotherapy, induced apoptosis in nonsmall cell lung cancer (NSCLC) cells via suppressing the formation of ROS [57]. Similarly, resveratrol enhanced the* in vivo* antitumor effects of 5-fluorouracil in mice inoculated with H22. Resveratrol instigated S phase arrest of H22 cells and also ameliorated the toxic effects of 5-FU [58].

#### 5.1.9. Resveratrol and Metastases

A phase I randomized double-blind clinical trial involving the administration of micronized resveratrol (SRT501) for 14 days to patients with hepatic metastases found resveratrol to be well tolerated, detectable in hepatic tissue, and also found significantly increased levels of caspase-3 in malignant hepatic tissue, suggesting resveratrol-mediated apoptosis of metastasized cancer cells [3]. Further evidence for resveratrol-mediated amelioration of metastases was reported by Brown et al. They documented that resveratrol treatment in 40 healthy volunteers for 29 days resulted in a reduction of circulating insulin-like growth factor-1 (IGF-1) and IGFBP-3 in all volunteers. Increases in IGF-1 and IGFBP-3 had been associated with metastasis, leading the authors to suggest that resveratrol may serve as a potential chemotherapeutic adjuvant [59].

*In summary*, several studies using animal models and different cancer cells* in vitro* have indicated that resveratrol appears to have several antitumor mechanisms. Considering similarities in the origin, pathogenesis, and progression of carcinomas, the tumor suppressing activity of resveratrol suggests the targeting of similar intracellular oncogenic molecular patterns by this phenolic phytochemical. Compared to these positive effects, the data from the limited human clinical trials has sown inconsistent outcomes of resveratrol supplementation. It should be noted that most of these clinical trials have small patient sample size and used different doses and different routes of resveratrol administration. It is worthy to note that some reports have shown adverse effects of resveratrol in certain cancer patients. For example, in a phase II clinical trial using a dose of 5 g/day in myeloma patients, resveratrol was found to cause nausea, diarrhea, and nephrotoxicity which may have led to one death [60]. Together, both* in vitro* and* in vivo* data suggest a need for more extensive and large clinical trials to emphasize the efficacy and safety of resveratrol as an anticancer agent. Currently, the most promising use of resveratrol is mostly as a cancer chemopreventive agent.

## 6. Resveratrol and Metabolic Disorders

Obesity, insulin resistance, and disorders of lipid metabolism are multifactorial diseases that may develop due to both intrinsic and extrinsic factors. Due to the complicated etiology and pathogenesis of metabolic disease, in many instances, therapy requires both behavioral modification and combination therapy, in addition to the use of dietary adjuvants which allow pharmacological synergism and an enhanced physiological response. Resveratrol, being a dietary constituent is an excellent candidate for adjuvant therapy for disorders with metabolic origin.

Since several studies using animal models of diet-induced obesity have shown beneficial effect of resveratrol on improving insulin sensitivity and obesity, many clinical trials were performed to speculate its effect as an antidiabetic agent in humans. For example, Brasnyó et al. evaluated the effects of 10 mg resveratrol supplementation for 4 weeks in a randomized, double-blind, placebo-controlled study involving patients with type 2 diabetes. The results of this this study reveal that resveratrol was effective in attenuating insulin resistance that was shown by a reduction in blood glucose levels and a delay in the time for peak postprandial glucose in patients receiving resveratrol compared to placebo [61]. Similarly, Labbé et al. demonstrated a positive effect on insulin sensitivity and blood glucose levels in mice following resveratrol treatment. Furthermore, resveratrol treated mice had a 33% reduction in inguinal fat in comparison to the control, even though the daily food and water intake was similar between the two groups [62].

Resveratrol supplementation significantly improved glycemic control, in clinical trials involving patients with impaired glucose tolerance. Resveratrol was effective in reducing hemoglobin-A1c levels, postprandial glucose levels, and postprandial insulin AUC by approximately 18% [63, 64].

Several studies have been conducted to investigate the mechanisms responsible for eliciting the positive blood glucose regulating effects of resveratrol. For example, Um et al. administered resveratrol to AMPK deficient mice and control mice for 13 weeks. Although resveratrol improved insulin sensitivity, metabolic rate, and glucose tolerance in the control mice, it failed to evoke any effects in AMPK deficient mice. The results of this study suggested that resveratrol mediated its metabolic effects primarily through activating AMPK [65]. Other postulated mechanisms include anti-inflammatory, antioxidant, and SIRT1-dependant suppression of protein-tyrosine phosphate 1B that acts on insulin receptors.

Resveratrol was also shown to exhibit favorable effects in obese mice on an atherogenic diet by ameliorating steatohepatitis via modulating the genes involved in lipid metabolism [66]. In a study by Gómez-Zorita et al., resveratrol decreased both whole body and liver weight in obese rats through reducing oxidative stress [67]. Although resveratrol administration to rodent models elicited positive effects on metabolic syndrome, resveratrol supplementation in obese men and nonobese postmenopausal women did not yield any significant metabolic benefits [68, 69].

Poulsen et al. reported a randomized, placebo-controlled clinical trial involving high-dose supplementation of resveratrol for obese men [68]. Specifically, following resveratrol administration, the substrate metabolism, insulin sensitivity, and body composition of 24 obese but otherwise healthy men enrolled in the 4-week long clinical trial were evaluated. Interestingly, the group did not notice an improvement in any of the study parameters, which may be attributed to the specific formulation of resveratrol used for administration. We contend that, given the pharmacokinetics of resveratrol, there may have been dramatic fluctuations in its bioavailability, a parameter that did not appear to be controlled across the enrolled group. Further, ethnicity data, which may affect pharmacogenomic variance, are not reported in their study and may contribute a cause for deviation from expected results [68].

Yoshino et al. conducted a similar study in nonobese Caucasian postmenopausal women with normal glucose tolerance, where they tested the effect of resveratrol administration for 12 weeks on various metabolic parameters in this study population. Additionally, they also investigated the effect of resveratrol on selected signal transduction molecules, such as Adenosine monophosphate kinase (AMPK), which is known to be activated in response to resveratrol treatment, but found no changes in any of the selected second messengers. However, in addition to the variability in the bioavailability of resveratrol as explained for the clinical study above, resveratrol was administered with food, which may lead to lower absorption [69]. The study conducted by this author group also suffered from another design drawback which may explain the lack of differences in the induction of signal transduction molecules. Of note is the fact that the biological samples were collected from skeletal muscle biopsies and subcutaneous adipose tissue harvests from all individuals enrolled in the study. Since the authors only examined signal transduction molecules from these limited tissue type, it is conceivable that the physiological effect of resveratrol was overlooked.

Thus, although both clinical trials indicate a paucity of resveratrol-mediated profound effects on metabolic markers such as insulin sensitivity, blood pressure, basal metabolic rate, plasma lipids, or body weight, the study design in both cases did not take into consideration some of the flaws discussed above.

In contrast, Dash et al. evaluated the effects of resveratrol administration for 2 weeks in obese men and reported a decrease in intestinal and hepatic lipoprotein particle production [70]. It is well established that elevated levels of hepatic apolipoprotein B (apoB-100) and intestinal apoB-48 particles are linked to the pathogenesis of hypertriglyceridemia [70]. Resveratrol's ability to modulate the production of intestinal and hepatic lipoproteins can alleviate hypertriglyceridemia.

Dal-Pan et al. postulated resveratrol's facilitation of weight loss in non-human primate models of obesity by increasing metabolic rate and suppressing torpor expression. In fact, resveratrol administration in primate models led to a 13% reduction in energy intake while increasing resting metabolic rate by 29% [71]. This study is further supported by Timmers et al. where, in a randomized, double-blind crossover study, resveratrol supplementation at 150 mg/day in 11 obese men mimicked the effects of caloric restriction by significantly reducing intramyocellular lipid content, hepatic lipid synthesis, blood glucose levels, and triglycerides in the subjects. The effects were linked to mechanisms involving increased AMPK, SIRT1, PGC-1∝ protein levels, and citrate synthase activity [72].

It is noteworthy that hypoglycemia, one of the major adverse effects associated with most of the antidiabetic agents, has not been reported with resveratrol in any animal or human studies, making resveratrol a viable option once its efficacy is thoroughly demonstrated.

## 7. Resveratrol and Cardiovascular Disease

One of the principal protective effects of resveratrol is on the cardiovascular system, where its use improves a variety of cardiac and hemodynamic functions. In fact, discovery of its cardioprotective effects was the primary reason that augmentation of therapy with resveratrol became a well-accepted notion.

Cao et al. reported resveratrol to mediate antihypertensive effects in angiotensin II induced hypertensive mice via activating AMPK and reducing expression of RhoA/ROCK genes. Furthermore, in spontaneously hypertensive rats, resveratrol treatment decreased blood pressure in comparison to control rats and ameliorated the progression of hypertension. These antihypertensive effects are linked with mechanisms involving the prevention of eNOS uncoupling and NO scavenging [73, 74].

Interestingly, resveratrol appeared to orchestrate multiple cardiac responses by a direct effect on cardiac function and restructuring. Kanamori et al. reported that high dose resveratrol improved cardiac performance via the AMPK pathway. In their experiments, resveratrol reduced left ventricular end-diastolic pressure and dilation, while preserving systolic pressure. In another study involving hypertensive rat models, resveratrol supplementation at 18 mg/kg/day for 8 weeks resulted in improved survival rate, reduction in HDL and LDL cholesterol levels, and reduction in markers of cardiac remodeling such as atrial natriuretic factor (ANF) [75]. These effects were linked to the upregulation of PGC-1a and PPAR∝ cardiac expression [76]. Resveratrol was also found to attenuate cardiac hypertrophy in another study of hypertensive rats through modulating the LKB1/AMPK signaling pathway [77].

Resveratrol mitigated platelet aggregation* in vitro* by reducing thromboxane A2 receptor levels, phospholipase C activity, and GPIIb/IIIa expression [78, 79]. The inhibitory effects of resveratrol on platelets were also evident* in vivo* through increased cGMP and Nitric Oxide (NO) levels. Activation of NO inhibited phospholipase C [80, 81]. The cardiovascular protective effect of resveratrol was further demonstrated in high-risk cardiac patients with aspirin resistance, where resveratrol significantly attenuated aspirin-resistant platelet aggregation, while exhibiting minimal effects on aspirin sensitive platelets [82].

The ischemia-reperfusion model has emerged as an excellent experimental system to validate the effects of various therapies on the heart and to elicit the molecular biology of the process. Pretreatment of rats with resveratrol followed by experimentally induced ischemia reperfusion, led to significant cardioprotection as evidenced by attenuated NOS (nitric oxide synthase) induction, decreased myocardial infarct size, and reduced numbers of cardiomyocytes. Resveratrol elicited cardioprotective effects through mediating NO-dependent pathways and inducing HO-1 expression, which involved p38 MAP Kinase and the PI-3 Kinase signaling systems [83, 84]. In separate experiments, resveratrol administration in rats improved ischemia-reperfusion injury through reduced calcium overload as a direct consequence of the inhibition of the Wnt5a/Frizzled-2 pathway. It also improved postischemic ventricular function via upregulated superoxide dismutase and peroxidase activities and downregulated catalase enzyme activity [85, 86]. Its cardioprotective effects were further validated in postmyocardial infarction patients, where resveratrol improved left ventricular ejection fraction and reduced LDL levels [87].

Resveratrol supplementation at 3 mg/kg/day in a hypercholesterolemic rabbit model led to a reduction in size, density, and mean area of atherosclerotic plaques and a decrease in the intimal layer thickness [88]. Resveratrol and a resveratrol containing mixture also elicited antiatherogenic properties in apoE-deficient mice. The mechanisms for resveratrol mediated antiatherogenic effects include the reduction of chemokines such as MCP-1 and the downregulation of intracellular adhesion molecule-1 (ICAM-1) and of vascular cell adhesion molecule-1 (VCAM-1) in the atherosclerotic vessels [89, 90].

In conclusion, resveratrol treatment provides far ranging cardiovascular protection by improving both cardiac function and architecture, while decreasing vascular effects such as platelet clumping, ischemia-reperfusion injury, and atherosclerosis. Several direct and indirect molecular targets mediating these cardiovascular effects have been identified and include AMP-activated protein kinase, cyclooxygenase 1, thromboxane A2, ICAM, NF-*k*B, and SIRT-1.

**Figure 2:**
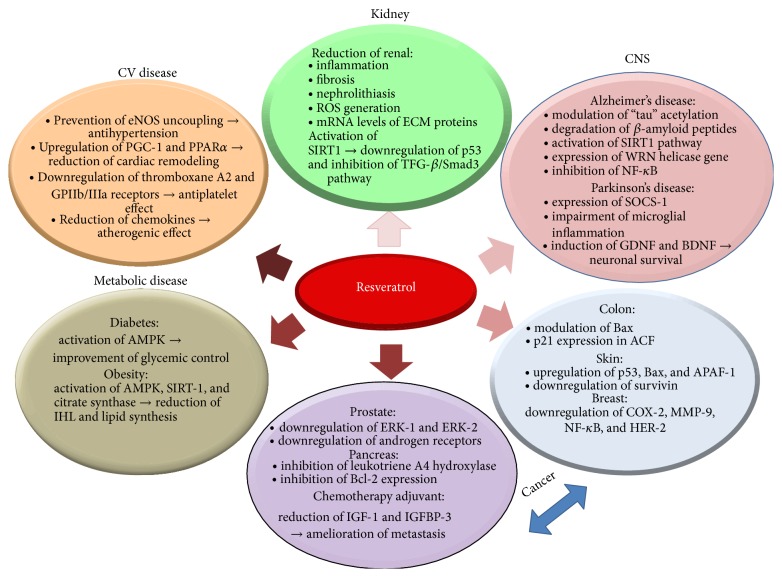
A summary of the published health benefits of resveratrol with the potential underlying molecular mechanisms. (See the Abbreviations section for complete definition of the abbreviations.)

In brief, plethora of reports from* in vitro* and laboratory animal studies in the past decade has suggested that resveratrol has considerable potential for therapy and/or protection for numerous acute and chronic diseases. These data were supported, in most part, by the emerging results from limited human clinical trials. Larger controlled human clinical trials are still needed to investigate dose- and formulation-dependent efficacy and safety of resveratrol and to study the effect of long-term effect of resveratrol supplementation.  Figure 2 provides a summary of the aforementioned health benefits of resveratrol with potential underlying molecular mechanisms.

## 8. Pharmacokinetics of Resveratrol

All the studies discussed thus far suggest that inclusion of resveratrol in therapy augments health benefits through multiple mechanisms of action. However, an important question that remains to be answered before translating* in vitro* and preclinical research into therapeutics is a consideration of the dose of resveratrol that would yield optimal response. Resveratrol doses that have been utilized in most of the published studies in animal models range from 0.1 to 1000 mg/kg. To add to the complexity, dose-dependent effects of resveratrol have been observed. For example, although high dose (400 mg/kg/day) causes a significant weight loss in mice, low dose (5 mg/kg) was found to induce weight gain [91]. Understanding the pharmacokinetics of resveratrol may significantly help answering this question.

Pharmacokinetic studies in human and animal models indicate that resveratrol bioavailability shows a high interindividual variation, which may limit its clinical application. The main reason found to contribute significantly to such variability is the role of intestinal microbial flora in resveratrol metabolism [92]. Although studies in rats report that 15–50% of orally ingested resveratrol is bioavailable, higher bioavailability (20–70%) was reported in human studies [93–95]. Comparison of bioavailability after a single oral dose to daily dose for 14 days did not show any evidence of saturation-limited absorption [95], suggesting perhaps that the uptake of resveratrol does not depend on a receptor-mediated process. Achieving adequate and consistent bioavailability at the desired doses is generally the major issue limiting future therapeutic application of resveratrol.  Table 1 summarizes strategies that have been utilized to enhance resveratrol bioavailability after oral administration.

Interestingly, dietary constituents were found to affect the rate and extent of resveratrol absorption. High fat meal significantly decreased the exposure level of resveratrol, as indicated by AUC and *C*_max⁡_ [96]. Although rapid uptake of resveratrol in humans resulted in a peak plasma concentration at 0.5 hours, administration under fasting conditions delayed the peak to 1.5–2 hours [93, 94]. It should be noted that none of these studies have included control groups where subjects administered resveratrol by itself; therefore, it may be difficult to conclude whether high fat meal or fasting condition significantly affects AUC and *C*_max⁡_ compared to resveratrol alone.

Resveratrol was quickly metabolized, with an average elimination half-life of 130–180 minutes, with glucuronide and sulfate conjugates, resulting in mixed sulfate-glucuronides, which were the primary metabolites [95]. It was suggested that these phase II conjugates underwent enterohepatic recycling where they got secreted back to the intestine, deconjugated, and subsequently either reabsorbed or excreted in feces [93]. Such extensive metabolism and rapid elimination of its conjugated metabolites may explain the low bioavailability of resveratrol.

Modulation of drug-metabolizing enzymes by resveratrol has been extensively studied* in vitro* and* in vivo*. Inhibition of benzo[a]pyrene-induced carcinogenesis by resveratrol was attributed to the ability of the latter to inhibit CYP1A1, an enzyme that is involved in the bioactivation of many carcinogens [97]. Administration of 1 g resveratrol for 4 weeks to forty-two healthy volunteers was found to inhibit the activity of CYP3A, CYP2D6, and CYP2C9 and to induce the activity of CYP1A2 [98]. As these P450 enzymes metabolize more than 75% of the drugs on the market, coadministration of resveratrol may influence the pharmacokinetics of drugs that are significantly metabolized by these enzymes. The same study revealed that resveratrol intervention induced the activity of phase II enzymes, especially glutathione-S-transferases (GST) in patients with low baseline enzyme activity. As phase II enzymes mediate detoxification of reactive metabolites and carcinogens, induction of this pathway by resveratrol could explain the chemopreventive effects described in the preceding sections. Comparison of the metabolism kinetics of resveratrol after a single dose and chronic administration provided no clear evidence of nonlinear kinetics [99].  Table 2 summarizes the average pharmacokinetic parameters for resveratrol based on limited published data from animal and human studies [94, 95, 100, 101].

## 9. Conclusion

Resveratrol affects multiple cellular processes and is an excellent candidate for use in human disorders. However, its physicochemical properties, coupled with its vagrant metabolism, may limit its bioavailability necessitating the design and fabrication of novel pharmaceutical delivery systems.

## Figures and Tables

**Table 1:** Approaches to enhance resveratrol's bioavailability.

Strategy	Examples	Effect on absorption pharmacokinetics	Reference
Solubility	(1) SRT501 formulation: a mixture of micronized resveratrol with particle size <5 *μ*m, flavors, colors, and emulsifiers. (2) Using hydroxypropyl-*β*-cyclodextrin.	More than 4-fold increase in *C*_max⁡_, AUC and 2-fold increase in *T*_max⁡_. Significant increase in rate of absorption with a negligible impact on bioavailability.	[3] [4]

1st-pass effect	(1) Coadministration with piperine, a polyphenol from black pepper. (2) Coadministration with quercetin, a plant flavonoid with potent antioxidant action.	1000% increase in *C*_max⁡_ and delaying the major glucuronide metabolite. Quercetin reduces the rate of resveratrol sulfate conjugation, but with no significant improvement on *C*_max⁡_ and AUC.	[5] [6]

Resveratrol precursors/prodrugs	Acetylation of the hydrophilic hydroxyl groups on resveratrol that are the major targets for sulfation and glucuronidation.	A significant increase in *C*_max⁡_ and AUC in a single animal study.	[7]

Nanoformulation	(1) Lipid-core nanocapsules.	Nanoformulation improves resveratrol's solubility, decreases gastrointestinal damage, and enhances distribution to tissues.	[8]
	(2) Bovine serum albumin nanoparticles.		[9]
	(3) Loaded solid nanoparticles.		[10]

**Table 2:** Average resveratrol pharmacokinetic parameters in both rats and human.

Bioavailability		Volume of distribution		Systemic clearance		Elimination half-life	
*F* (%)		*V*_*d*_ (L/kg)		*Cl* (mL/min/kg)		*t*_1/2_ (min)	
Rats	Human	Rats	Human	Rats	Human	Rats	Human
29	70	35	30	184	157	130	180

## Abbreviations

ACF: Aberrant crypt foci
AD: Alzheimer's disease
AKI: Acute kidney injury
ALS: Amyotrophic lateral sclerosis
AMPK: Adenosine monophosphate kinase
APAF-1: Apoptotic protease activating factor-1
ANF: Atrial natriuretic factor
APAF-1: Apoptotic protease activating factor-1
APC: Adenomatous polyposis coli
ApoB: Apolipoprotein B
AUC: Area under the curve
BCL-2: B-cell lymphoma 2
BDNF: Brain derived neurotrophic factor
BFGF: Basic fibroblast growth factor
cAMP: Cyclic adenosine monophosphate
cGMP: Cyclic guanosine monophosphate
CREB: cAMP-response-element-binding protein
DMBA: Dimethylbenz[*α*]anthracene
ECM: Extracellular matrix
eNOS: Endothelial nitric oxide synthase
ERK-1: Extracellular regulating kinase 1
GDNF: Glial cell line-derived neurotrophic factor
GST: Glutathione-S-transferases
HCC: Hepatocellular carcinoma
HDL: High-density lipoprotein
HER-2: Human epidermal growth factor 2
HO-1: Heme-oxygenase-1
H22: Hepatocarcinoma cells 22
ICAM-1: Intracellular adhesion molecule-1
IGFBP-3: Insulin-like growth factor binding protein-3
ILC: Intramyocellular lipid content
ILGF-1: Insulin-like growth factor-1
LDL: Low-density lipoprotein
LKB1: Liver kinase B1
MAP: Mitogen-activated protein
MCP-1: Monocyte chemoattractant protein-1
MDA: Malondialdehyde
MMP-9: Matrix metaloproteinase-9
MPP^+^: 1-Methyl-4-phenylpyridinium
NFR2: Nuclear factor erythroid factor
NF*k*B: Nuclear factor kappa B
NFT: Neurofibrillary tangles
NOS: Nitric oxide synthase
NSCLC: Nonsmall cell lung cancer
PD: Parkinson's disease
PPAR-*γ*: Peroxisome proliferator-activated receptor gamma
ROCK: Rho-associated, coiled coil containing protein kinase
ROS: Reactive oxygen species
SERM: Selective estrogen receptor modulator
SIRT-1: Sirtuin 1
SOCS-1: Suppressor of cytokine signaling 1
STAT: Signal transduction and activator of transcription
TGF-*β*: Transforming growth factor beta
TLR4: Toll-like receptor 4
TNF-*α*: Tumor necrosis factor alpha
VCAM-1: Vascular cell adhesion molecule-1
VEGF: Vascular endothelial growth factor
4E-BP1: 4E-binding protein 1
5-FU: Fluorouracil
8-OHdG: 8-Hydroxydeoxyguanosine.
